# Bird use of organic apple orchards: Frugivory, pest control and implications for production

**DOI:** 10.1371/journal.pone.0183405

**Published:** 2017-09-14

**Authors:** Anna M. Mangan, Liba Pejchar, Scott J. Werner

**Affiliations:** 1 United States Department of Agriculture, Animal and Plant Health Inspection Service, Wildlife Services, National Wildlife Research Center, Fort Collins, Colorado, United States of America; 2 Department of Fish, Wildlife, and Conservation Biology, Colorado State University, Fort Collins, Colorado, United States of America; Auburn University, UNITED STATES

## Abstract

As the largest terrestrial biomes, crop and pasturelands can have very large positive or negative impacts on biodiversity and human well-being. Understanding how animals use and impact agroecosystems is important for making informed decisions that achieve conservation and production outcomes. Yet, few studies examine the tradeoffs associated with wildlife in agricultural systems. We examined bird use of organic apple orchards as well as how birds influence fruit production positively through control of an economically important insect pest (codling moth (*Cydia pomonella*)) and negatively through fruit damage. We conducted transect surveys, observed bird frugivory and assessed bird and insect damage with an exclosure experiment in small organic farms in western Colorado. We found that organic apple orchards in this region provide habitat for a large number of both human-adapted and human-sensitive species and that the species in orchards were relatively similar to adjacent hedgerow habitats. Habitat use did not vary as a function of orchard characteristics, and apple damage by both birds and *C*. *pomonella* was consistent within and across apple blocks that varied in size. A small subset of bird species was observed foraging on apples yet the effect of birds as agents of fruit damage appeared rather minor and birds did not reduce *C*. *pomonella* damage. Our results demonstrate that organic apple orchards have the potential to provide habitat for diverse bird communities, including species typically sensitive to human activities, with little apparent effect on production.

## Introduction

More than 40% of the land on Earth has been converted to agriculture or urban development, and much of the undisturbed habitat that remains is fragmented by infrastructure associated with these land uses [1]. Crop and pasturelands are the largest terrestrial biomes and land use change associated with agriculture is the leading driver of biodiversity loss worldwide [2]. Thus, these lands have the potential to have very large negative or positive effects on biodiversity and human well-being. Direct costs of increased agricultural production and landscape simplification include species extinctions, pollution [3], and unintended consequences for agricultural production through the loss or decline of populations of pollinators and natural enemies of insect pests [4]. Yet agriculture is critical for protecting global food security and supporting the economy of many nations [5]. Furthermore, agroecosystems provide co-benefits for biodiversity [6] and contribute to a variety of ecosystem services including carbon sequestration, regulation of soil and water quality, and cultural services [5]. As the demand for food increases with population growth and changing diet preferences, sustaining productive agroecosystems that also conserve biodiversity is more important than ever [3].

Ecological research in agricultural systems has focused largely on the detrimental impacts of agriculture on biodiversity and the “ecosystem disservices” or negative effects of animals on crop production. Meanwhile, the capacity of agroecosystems to support wildlife habitat and the potential benefits of those animals for food production remain understudied [7]. Research suggests that birds in particular can fill important roles in ecosystem function, as well as provide benefits to human communities such as pest control, seed dispersal, pollination and decomposition [8, 9]. Birds can also act as bio-indicators, providing early warning of environmental change [10].

Organic fruit orchards are perfect systems for advancing our understanding of the services and disservices that birds can provide, as well as evaluating the capacity of agroecosystems to provide habitat for diverse animal communities. Fruit damage by wild birds can have serious economic impacts on producers [11], yet in return, birds can deliver pest control services in orchards making small, yet valuable impacts on both insect numbers and fruit yield [12]. Relatedly, farms can vary in their capacity to provide quality habitat for bird communities depending on their size, plant diversity, management practices and surrounding land cover [13, 14].

In western Colorado (USA), organic apples (*Malus domestica*) are an important cash crop that appear to provide habitat for birds. Yet, the composition of the bird community and the degree to which individual species cause damage to the fruit or play an active role in arthropod pest mitigation is unknown. Although anecdotal observations by farmers suggests that wild birds are capable of causing fruit damage, the codling moth (*Cydia pomonella*) is considered the most economically important pest for apples in this region. Adult moths emerge in spring, lay eggs directly on apples and leaves, and the larvae burrow into the apples to feed on the developing fruit and seeds when they hatch [15]. Fully developed larvae move to the bark or soil to spin cocoons and pupate. There are typically three generations of *C*. *pomonella* each year in western Colorado, with the last generation overwintering as pupae to reemerge in spring [15]. *C*. *pomonella* are capable of causing extremely high levels of damage, up to 90% of the fruit crop, if left uncontrolled [16]. Organic farmers use an integrated pest management (IPM) approach including organic pesticides, pheromone treatments, and varied cultural practices in an attempt to limit damage. These IPM approaches can be costly and labor intensive [17]. As insectivorous predators, some birds may be contributing to pest insect reduction, including *C*. *pomonella*, and thus providing a valuable service to organic farmers [18]. Although it has long been suggested that the pest control service provided by birds outweighs the damage that they cause [19], few studies have quantified this potential tradeoff while also documenting the extent to which agroecosystems provide habitat for diverse bird communities.

To fill this knowledge gap, our primary objectives were to: 1) determine how bird species richness and composition differ between apple orchards and adjacent habitat, and what factors influence habitat use by particular species within orchards; 2) identify which bird species depredate fruit; and 3) evaluate the magnitude of fruit damage and pest control in this system. We conducted bird surveys and foraging observations in and directly adjacent to apple orchards, and assessed bird and *C*. *pomonella* damage using an exclosure experiment to achieve the objectives of this study.

## Materials and methods

### Study region

**Fig 1:**
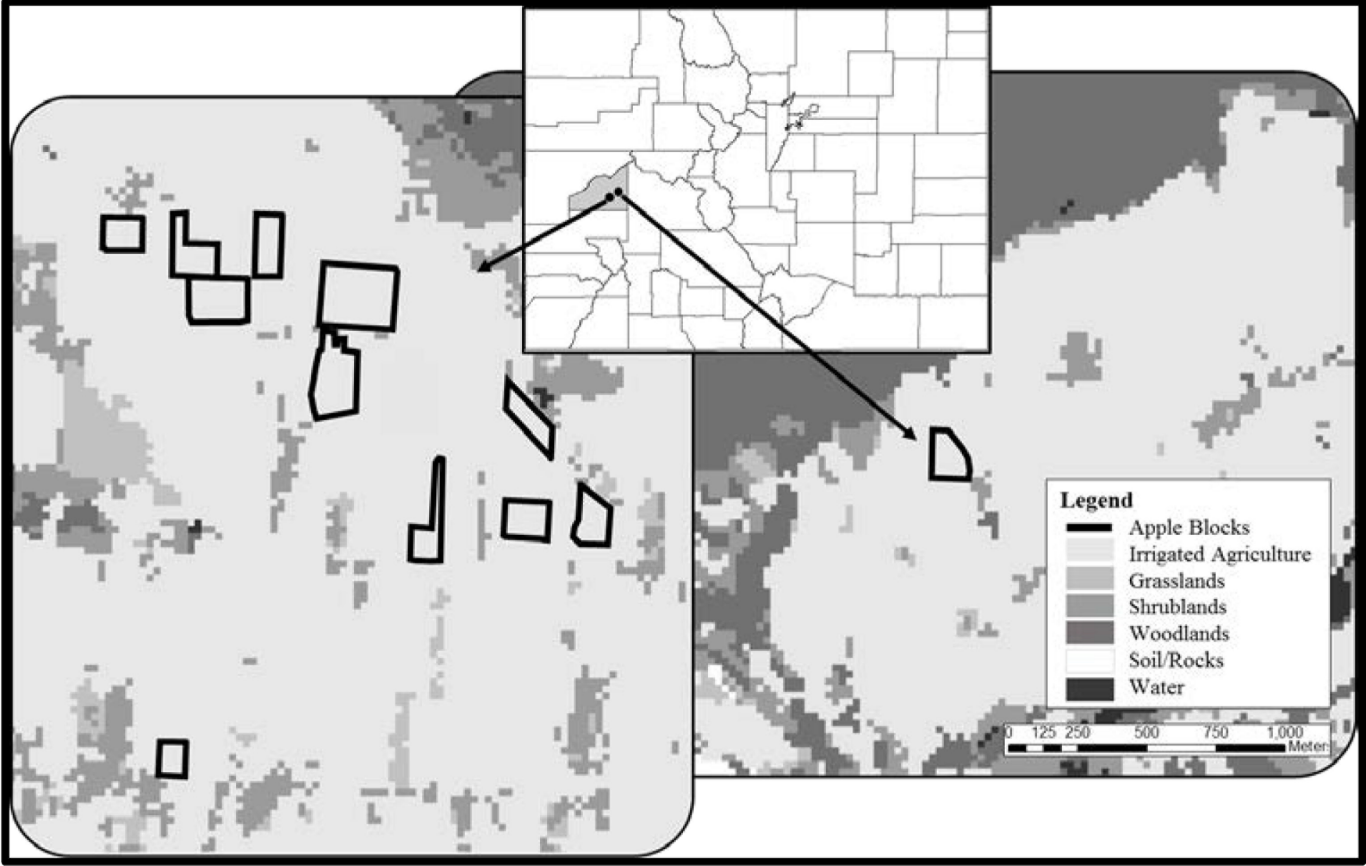
Study area. Study site locations in Delta County, Colorado, USA (grey-shaded polygon on Colorado map). Black-lined polygons indicate apple blocks studied in the towns of Hotchkiss (left) and Paonia (right). Land cover vegetation classes are illustrated in grey scale at a resolution of 25 meters [21]. Irrigated agriculture includes fruit orchards, vegetable crops, hops and hay. Other major land cover types in this region include grasslands (i.e., annual and perennial grasses), shrublands (e.g., Gambel oak (*Quercus gambelii*), serviceberry (*Amelanchier spp*.) and saltbrush (*Atriplex spp*.)) and woodland (e.g., pinyon (*Pinus edulis*)-juniper (*Juniperus osteosperma*), aspen (*Populus tremuloides*) and spruce (*Picea spp*.)). Roads, property boundaries, etc. are not included to protect the anonymity of collaborating farmers.

This study was completed in partnership with three organic fruit orchards in Delta County, Colorado (38°51'00"N, 107°45'00"W, Fig 1), which consists of nearly 24,000 ha of irrigated agriculture. More than 750 ha in this county are dedicated to orchards growing a variety of fruit crops including blocks of apples, peaches, cherries and grapes. There are 91 farms growing a total of 244 ha of apples and the average farm size is 81 ha (median = 15 ha) [20].

### Study design

Ecological research was conducted on farms that were representative of organic fruit orchards in Delta County in terms of size, crops grown and general management practices. We reached out to all 16 farmers growing apples in the county who had publicly available contact information to request access to potential study sites. Participating farms were selected based on three criteria: 1) informal conversations with owners indicating observed damage from bird and insect pests, 2) minimal use of bird control techniques and pesticides (i.e., USDA organic), and 3) farmer willingness to participate. All three farms that met the above criteria were included in the study. Average size of the participating farms was 52.86 ha (range 25.04–88.70 ha).

We surveyed all apple blocks in participating farms that were large enough to conduct the methods described below, where apple trees were producing fruit, and where access was granted. The average apple block was 2.78 ha (range 1.19–5.99 ha) and contained 2–3 apple varieties ($\bar{x}$ = 2.3, range 1–6); Gala and Honeycrisp were the predominant varieties. All sampling occurred during the 2015 growing season (i.e. fruit set to harvest; June–October), although some data were collected in a subset of apple blocks or months, as described below. Previous research suggests that birds primarily use the outer portions of crops [22]; we therefore used a stratified study design to distinguish between the interior and edges (i.e., outermost 18 m) of a block. While various distances have been examined as thresholds between interior and edge rows of crops (i.e. [23, 24]), we selected an 18 m threshold as this distance exhibited a visual barrier to the adjacent habitat and because interior/edge damage estimates from other crop studies have been statistically different at this distance [25, 26]. Apple blocks were embedded in a diverse landscape of agricultural crops and uncultivated vegetation; consequently the type of habitat directly adjacent to the edges varied. Therefore, sampling within edges was further stratified into three categories of adjacent habitat within study orchards: crop (i.e., peach and cherry), grass (i.e., irrigated hay and fallow fields) and shrub/tree (i.e., vertical vegetation such as hedgerows).

### Bird species richness, similarity and habitat use

Since this study system is substantially altered by humans (Fig 1), we anticipated that birds more adapted to human-dominated landscapes would use the area more frequently than those sensitive to anthropogenic land uses. To differentiate among these groups, we classified bird species as human-adapted if previous studies reported that occupancy/use, activity or abundance/density stayed the same or increased with urbanization or housing density, a proxy measure for human disturbance ([27]; S1 Table). Species were classified as human-sensitive if decreases in these relationships were reported. These classifications provide a useful means to investigate the value of apple orchards for ubiquitous birds as well as those of greater conservation concern in a human-dominated landscape.

Bird surveys were conducted in twelve apple blocks in June–September 2015 using line transect surveys (n = 38 transects) to quantify species richness, composition and habitat use by human-adapted and human-sensitive species. Transects were 50 m or 100 m long depending on the size and shape of the surveyed apple block and were located in the interior (n = 19) and along the edges of apple blocks (n = 19). Transects less than 150 m apart were surveyed on consecutive days. Standard protocol [28] was used to survey each transect up to seven times ($\bar{x}$ = 6.3 ± 0.1). Two observers recorded all birds detected aurally or visually, where each bird was detected (i.e., interior, edge or adjacent habitat) and the distance to each bird was estimated with the assistance of a laser rangefinder (Bushnell Sport 850 or Bushnell Yardage Pro 1000). Observers were trained to walk at a similar slow and steady pace to help ensure equal sampling effort, but were allowed to stop and observe birds as needed to confirm species identification [29]. Survey and site-specific covariate data were also collected (S2 Table). Surveys were conducted between dawn and four hours after sunrise in good weather (i.e., no rain or high winds) and were not conducted during the application of organic pesticides or the four-hour re-entry period following their application. These observational methods were employed on privately owned land and were approved by the United States Department of Agriculture, Animal and Plant Health Inspection Service, Wildlife Services, National Wildlife Research Center’s Institutional Animal Care and Use Committee (QA-2286).

### Bird and *C*. *pomonella* damage

Behavioral observations were conducted in ten apple blocks in July–September 2015, when the developing fruit was more apt to receive bird damage (AMM personal observation). Observations were carried out in a subset of apple blocks to identify the relative importance of frugivorous species [30]. The block shape (e.g. rectangle or irregular polygon) and adjacent vegetation were not consistent across apple blocks so we conducted observations in the interior of 8 blocks and along 14 edges of 9 blocks (i.e. cherry = 3 edges, peach = 4, grass = 3, shrub/tree = 4). Four observers began each observation session in either the interior or near the edge of an apple block and used a digital recorder (Olympus) to record each species and the number of apples that the bird damaged. A time restriction (i.e. 30 min) was used to standardize effort across the unequal areas of the observation sessions. A total of 226 surveys were conducted during the morning (sunrise to 10:30 h), afternoon (approximately 12:00 to 14:00 h) and evening (approximately 18:00 to sunset) for 113 hours of field observations.

**Fig 2:**
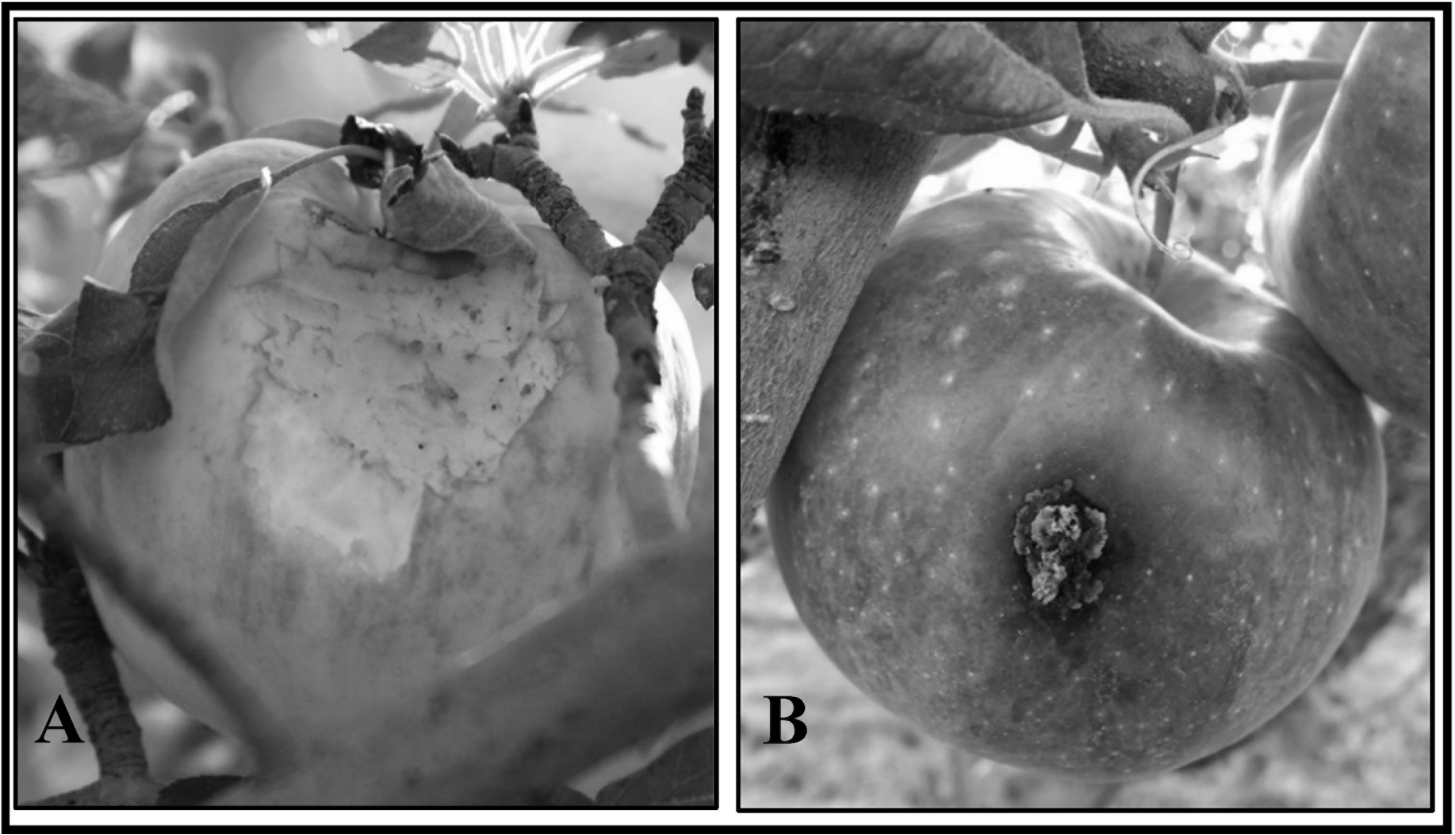
Bird and *C*. *pomonella* damage. Characteristic damage to organic apples in Delta County, Colorado. (A) Bird damage is identified by v-shaped peck marks and subsequent scrapes on the skin and flesh of the fruit, and (B) *C*. *pomonella* damage is characterized by stings (i.e., larval entry points) and frass (i.e., brownish excrement).

To comparatively assess apple damage by birds and damage by *C*. *pomonella* in the presence and absence of birds, we installed netted bird exclosures on 55 trees after fruit set (July 2015) in eight apple blocks in the interior (n = 23) and along the edges (crops n = 16, grass n = 8, shrub/tree n = 8). Bird exclosures consisted of 19 mm mesh plastic netting (Bird-X) and each enclosed 25 (± 10) apples. Another 55 unnetted control trees within 10 m of each paired exclosure tree were selected and 25 (± 10) apples were designated using colored flagging to denote the branch on which they were growing. Apples in the experiment were assessed for bird and *C*. *pomonella* damage up to five times ($\bar{x}$ = 3.3 ± 0.1) from July–October 2015. Only unnetted control trees were assessed for bird damage (Fig 2A). *C*. *pomonella* damage was identified by diagnostic markings on the surface of apples (e.g. stings and frass; Fig 2B).

### Statistical analysis

#### Bird species richness and composition

We created separate encounter histories of human-adapted and human-sensitive species in each habitat type from across all farms which included the total number of each species detected as well as the number of surveys conducted. Using these encounter histories, sample coverage, species richness estimates and confidence intervals were generated with program SimAssem [31]. We used the second-order jackknife estimator to compare species richness in apple blocks and adjacent habitat. Jackknife estimators reduce bias due to sampling effort and species’ rarity by subsampling from the entire sample to estimate the number of undetected species [32, 33]. To compare the composition between apple blocks and adjacent habitats, we calculated a Jaccard similarity index using the number of species detected in each habitat type for all pairwise combinations of habitat (i.e., apples, adjacent grass and adjacent shrub/tree).

#### Bird habitat use

We used single-season, single-species occupancy models [34] to test for differences in use of apple blocks by birds with package RMark [35] in R [36]. Because bird home ranges extend beyond the boundaries of the interior and edge transects, the model assumption that a sampling unit is closed to immigration and emigration is not met. We therefore consider our estimates of occupancy as the species’ probability of habitat use rather than true occupancy [37]. Occupancy models included main predictor variables of habitat use (i.e., farm management, habitat location, and size and edge-to-area ratio as calculated using ESRI [38]) as well as other survey and site covariates that were expected to influence detection (S2 Table). If transects were in the same habitat type but less than 200 m apart, we pooled all the detections into a single transect for analyses (n = 31). Detected birds were assigned to an appropriate transect (i.e., interior, edge crop, edge grass or edge shrub/tree; flyovers were excluded) which were collapsed into two main categories, interior (n = 15 transects) or edge of apple blocks (n = 16). All continuous predictor covariates were standardized (*x*—$\bar{x}$)/*σ*. For the purpose of avoiding over parameterization, one of each set of covariates with a correlation value greater than |0.7| was eliminated, or was included only in separate models when the covariates represented competing hypotheses [39]. For example, we included apple block size and edge-to-area ratio as predictor variables in our model set, but never in the same models because they were correlated.

We ran a preliminary analysis for each species to determine the model complexity that the data would support and all possible combinations of remaining biologically-meaningful models were built [40], including single, additive and two-way interactive effects. Models with variables that did not converge or could not be estimated were eliminated from the model set. Model selection was based upon Akaike’s Information Criterion for small sample size (AICc). We used a parametric bootstrap procedure [41] with 10,000 simulated datasets in the unmarked package [42] in R to assess model fit for each species using the most global model. For species that showed overdispersion in the data, the overdispersion correction parameter ($\hat{c}$) was used to adjust the model selection and calculate QAICc values. The most parsimonious models for each species were determined, and we assessed the strength and directionality of each occupancy covariate from the top model containing the covariate [39].

#### Bird and *C*. *pomonella* damage

Observations of birds feeding on apples were pooled by species to calculate the proportion of apples damaged by the total number of observations. For apple damage assessment data, we used a Kruskal-Wallis test to compare median bird damage as a function of location within the apple block (i.e., interior vs. edges) and apple variety because data did not meet normality assumptions (Shapiro-Wilk W = 0.66, p = 4.20e-10). Bird damage was assumed to be cumulative (i.e., the number of bird damaged fruits would only increase on subsequent assessments) therefore only the assessment visit with the greatest number of apples damaged per tree was used in the analysis. This selection of data for analysis accounted for the occasional bird-independent loss of fruit from the trees through time. Only Gala (20 trees) and Honeycrisp (23 trees) were included in the analysis as other varieties had sparse data (3 or fewer trees). The proportion of bird-damaged apples was arcsine transformed.

To investigate if birds reduced apple damage by *C*. *pomonella*, we quantified the damage as a function of bird access (i.e., netted treatment or unnetted control) as well as other apple block characteristics (S3 Table) using generalized linear mixed models with a logit link function and random effect for the apple block. Overdispersion was modeled as an observation-level random effect. Only treatment and control tree pairs that were well matched in terms of variety, height above the ground and habitat location (i.e., interior (n = 14 pairs) or edge (n = 21)) were included in this analysis. If apples inside exclosures showed signs of bird damage, it was assumed that birds could access insects within the netting and the pair was excluded from the analysis. There was a difference between our ability to measure *C*. *pomonella* damage on apples from control and exclosure trees that affected data handling. Because fallen apples in exclosures were caught in nets, we were able to track the fate of all apples, and subtract apples with damage at the time of installation from subsequent visits in the analysis phase. Because we did not know the damage status of fallen apples on control trees, we could not use this information to adjust future damage counts. *C*. *pomonella* damage was assumed to be cumulative, therefore only the assessment visit with the greatest number of apples damaged per tree was used in the analysis. All combinations of singular and additive effects were used to create a candidate model set. Variables that would not converge were excluded and pretending variables (i.e. location) were removed from the candidate model set. Model ranking was based upon AICc, and model averaging was used to assess the strength and directionality of predictor variables from information in the entire model set [39, 43] with package AICcmodavg [44] in R.

## Results

### Bird species richness, similarity and habitat use

During transect surveys and foraging observations 59 bird species were detected, including 40 human-adapted species and 19 human-sensitive species (S1 Table). The only species richness estimates without overlapping confidence intervals were between the grass and shrub/tree habitats for human-adapted species, and between the human-adapted and human-sensitive species within the shrub/tree habitat (Table 1). The species composition was most similar between apple blocks and adjacent shrub/tree habitat (Jaccard similarity index (S_j_) human-adapted: S_j_ = 0.64, human-sensitive: S_j_ = 0.73), followed by apple and adjacent grass habitat (human-adapted: S_j_ = 0.44, human-sensitive: S_j_ = 0.27) (S4 Table). Adjacent shrub/tree and adjacent grass habitats were least similar (human adapted: S_j_ = 0.39, human-sensitive: S_j_ = 0.24).

**Table 1:** Second-order jackknife estimates of species richness. Confidence intervals (95% CI) associated with richness estimates in the apple blocks overlapped those of the adjacent habitat types for both human-adapted and human-sensitive groups of birds.

	Species Richness Estimates (95% CI)	
	Human-Adapted	Human-Sensitive
**Apple Blocks**	35.79 (29.13, 53.19)	17.10 (13.32, 31.60)
**Adjacent Shrub/Tree**	47.64 (37.98, 67.71)	17.91 (14.77, 33.93)
**Adjacent Grass**	14.55 (14.01, 36.32)	14.88 (9.77, 29.29)

Of the 37 bird species detected in the apple blocks during transect surveys, seven had sufficient detections for occupancy modeling. Human-adapted species included the American robin (*Turdus migratorius*), Brewer’s blackbird (*Euphagus cyanocephalus*), cedar waxwing (*Bombycilla cedrorum*) and house finch (*Haemorhous mexicanus*). Human-sensitive species included the blue grosbeak (*Passerina caerulea*), lesser goldfinch (*Spinus psaltria*), and Lewis’s woodpecker (*Melanerpes lewis*). Habitat use (psi) was best explained by a constant occupancy structure (i.e., no covariates) on psi for six out of the seven species modeled; the edge vs. interior habitat location covariate was in the top model for lesser goldfinch (Table 2). Main predictor variables (i.e., management, habitat location, size and edge-to-area ratio) appeared in model sets with considerable empirical support (i.e., Δ(Q)AICc < 2) [39] for some species. Although these models explained substantial variation in the data, confidence intervals of the parameter estimates included zero, so we concluded that the effects of the parameters were weak and do not discuss them further. Detection probability (p) was best explained by a quadratic effect on time in four of the modeled species and as an additive effect with survey effort for one additional species (Table 2). Detection was best explained by a constant structure on p for the remaining two species.

**Table 2:** Top occupancy model sets. Models with strong support (i.e. Δ(Q)AICc < 2) are shown. AICc values were used in models with $\hat{c}$ values = 1 and QAICc values were used in models with $\hat{c}$ values > 1. Number of parameters (k), model weight (*w*_i_), log likelihood (-2LnL) and overdispersion parameter ($\hat{c}$) values are also reported.

Species	k	(Q)AICc	Δ(Q)AICc	*w*_i_	-2LnL	c-hat
Model						
**Human-Adapted**						
American robin						
p (Time^2) psi (.)	3	97.603	0.000	0.405	190.500	2.1
p (Time^2) psi (Edge-to-Area)	4	99.471	1.867	0.159	188.858	2.1
Brewer's blackbird						
p (Time^2) psi (.)	3	62.995	0.000	0.191	58.350	1
p (Time^2) psi (Edge-to-Area)	4	64.100	1.105	0.110	56.744	1
p (Time^2 + Effort) psi (.)	4	64.642	1.647	0.084	57.308	1
p (Time^2) psi (Size)	4	64.696	1.701	0.082	57.364	1
cedar waxwing						
p (.) psi (.)	2	62.089	0.000	0.084	57.660	1
p (Observer) psi (.)	3	62.293	0.204	0.076	55.404	1
p (.) psi (Size)	3	62.478	0.390	0.069	55.590	1
p (Observer) psi (Size)	4	62.969	0.881	0.054	53.431	1
p (.) psi (Edge-to-Area)	3	63.766	1.677	0.036	56.877	1
p (Effort) psi (.)	3	63.892	1.803	0.034	57.003	1
p (Time^2) psi (.)	3	64.086	1.997	0.031	57.197	1
house finch						
p (.) psi (.)	2	96.848	0.000	0.257	262.471	2.8
**Human-Sensitive**						
blue grosbeak						
p (Time^2) psi (.)	3	52.192	0.000	0.218	92.418	2
p (Observer) psi (.)	3	52.415	0.224	0.195	92.874	2
p (.) psi (.)	2	53.255	1.063	0.128	99.605	2
p (Time^2) psi (Size)	4	53.842	1.651	0.096	90.380	2
lesser goldfinch						
p (Time^2) psi (Location)	4	115.066	0.000	0.206	105.528	1
p (Observer) psi (Location)	4	115.299	0.233	0.184	105.760	1
p (Observer) psi (Management + Location)	5	115.407	0.341	0.174	103.007	1
Lewis's woodpecker						
p (Time^2 + Effort) psi (.)	4	81.064	0.000	0.252	71.526	1
p (Observer + Effort) psi (.)	4	81.742	0.678	0.180	72.204	1
p (Time^2) psi (.)	3	82.838	1.773	0.104	75.949	1
p (Time^2 + Effort) psi (Location)	5	82.894	1.830	0.101	70.494	1
p (Observer) psi (.)	3	82.982	1.918	0.097	76.094	1

### Bird and *C*. *pomonella* damage

Of the 21 bird species recorded in apple trees during foraging observations, only five were observed damaging fruit. Two species, Bullock’s oriole (*Icterus bullockii*) and Woodhouse’s scrub-jay (*Aphelocoma woodhouseii*), were observed infrequently (n = 4 and n = 1, respectively) and were therefore excluded from further discussion. The three species with sufficient observations (n > 5) and foraging events were the common raven (*Corvus corax*; n = 12), Lewis’s woodpecker (n = 35) and house finch (n = 129). The proportion of apples damaged by total observations of these species was 0.75, 0.69 and 0.12, respectively. For example, during 12 observations, common ravens damaged 9 apples. They were not foraging, or were feeding on other items, during the other 25% of observations.

Very few of the unnetted apples (2.3%, n = 1205) had measurable bird damage. There were no statistically significant differences between apple damage on interior trees or edges adjacent to crops, grass or shrub/tree (Kruskal-Wallis χ^2^ = 4.25, p = 0.24), nor between apple varieties (Kruskal-Wallis χ^2^ = 3.32, p = 0.07). Damage from *C*. *pomonella* was recorded on 7.5% (n = 1481) of all paired treatment and control apples assessed for damage. *C*. *pomonella* damage was greater in unnetted control trees than in treatment trees where birds were excluded. The model averaged estimate for the netting treatment indicated a statistically significant negative trend (β = -0.97 (95% CI = -1.61, -0.32)). However, the treatment effect was not the only predictor variable supported in our analysis; apple variety and farm management were both in models with considerable support (S5 Table). In addition to the treatment, Honeycrisp varieties and farm management group B had statistically significant negative trends in *C*. *pomonella* damage (β = -1.1 (-1.92, -0.27) and β = -1.46 (-2.24, -0.68), respectively).

## Discussion

This study demonstrates that both apple blocks and uncultivated shrub/tree habitat on organic orchards in western Colorado support bird activity by many species. Interestingly, this included species that commonly co-exist with human communities as well as species that are typically sensitive to human development. There was no evidence that birds reduce *C*. *pomonella* damage, but importantly, apple damage by birds was also minimal and associated with few bird species. These results suggest that diverse bird species use landscapes associated organic apple orchards with few apparent positive or negative effects on production.

With over 60% of bird species detected in apple blocks, this agroecosystem appears to provide habitat for a large number of species in the study region. While there was little overlap in the composition between apple blocks and adjacent grass habitat, there was high similarity between birds found in apple trees and adjacent uncultivated shrub/tree habitat. Trends in species richness showed a similar pattern with estimates in apple blocks higher than that of the adjacent grass but lower than the adjacent shrub/tree habitat. Interestingly, these trends did not differ between human-adapted and human-sensitive species, although the total number of human-adapted birds was greater and the trends were more pronounced. These findings, in a mosaic system of fruit blocks and adjacent strips of uncultivated vegetation within farm boundaries, are consistent with recent literature demonstrating that hedgerows can enhance avian diversity in field crops [45]. Our results suggest that the benefits of this management practice could be extended to birds in more structurally diverse systems such as orchards, even for some birds typically sensitive to human activities. For example, woodland birds such as the Lewis’s woodpecker, a Watch List species [46], may benefit from the structure and food resources provided by orchards and adjacent linear patches of shrubs and trees.

Habitat use for the modeled bird species in this system was not strongly associated with any of our predictor variables. It is possible that the species detected frequently enough to meet the requirements of the model may use all apple blocks often and indiscriminately. Additionally, apple blocks in this region are relatively similar in size and shape. Bird habitat use may vary in response to apple block size or edge-to-area ratio in landscapes with greater heterogeneity in orchard size and configuration.

The subset of birds that cause apple damage was previously unknown in this system. Because of the conspicuousness of birds and the high variability in the damage that they cause, objective damage estimates by producers can be difficult. Bird damage is often attributed to the most abundant or noticeable species and these observations can influence control decisions [30]. Before our study for example, damage was attributed to house finches by several farmers, as described during informal conversations. Yet, only 12% of our observations of this species included fruit depredation (n = 129). Of the five bird species observed damaging fruit, four were classified as human-adapted, but the Lewis’s woodpecker, a human-sensitive species, also caused damage. Direct observation, although effective for describing bird species depredating fruit, is not an effective method for identifying avian predators of *C*. *pomonella* [47]. Thus, we were unable to directly compare bird species damaging fruit and those consuming pests.

Although the proportion of foraging events was high for a few species, the minimal damage by a small subset of the bird community suggests that wild birds are not a major concern for organic apple producers in this region. Therefore, it would not be beneficial to exclude particular bird species from apple blocks. While these results are encouraging for apple production, other fruit crops in the region, such as cherries and grapes, experience higher reported levels of fruit depredation. As multiple fruit crops are often grown on the same farm, the variation in damage may affect the willingness of producers to promote avian diversity as a part of their agroecosystem, despite our findings that birds inflict little damage to apples. Scaling research up to multi-crop systems could provide more complete information on the services and disservices provided by birds.

Apple damage from *C*. *pomonella* was relatively low (7.5%), suggesting that this insect is being controlled reasonably well by current organic pest management practices. However, there is always concern for pesticide resistance or outbreaks that could have important economic consequences for producers [14]. Others have suggested that birds can provide additive services by extending the time between insect outbreaks [12] or providing additive pest control during outbreaks in some agroecosystems [48]. We were not surprised to find that differences in *C*. *pomonella* damage were related to farm management, as some farms input more organic products and labor to control the pest than others. We also anticipated that some varieties would experience more damage than others, and our results showed that Honeycrisp apples had more damage than Galas or other varieties. Contrary to our expectations however, we found less *C*. *pomonella* damage on apples protected from birds with netting, providing no evidence that birds were mitigating *C*. *pomonella* damage in this system. It is possible that the exclosure material (i.e., 19 mm plastic mesh) deterred *C*. *pomonella*. However, adult moths are typically 10–12 mm in length with a 15–20 mm wingspan [49], and furthermore, similar exclosure experiments have used even smaller mesh (i.e., 15 mm) and found significantly more *C*. *pomonella* damage within exclosures [18]. Differences between our results and other exclosure experiments may be due to different installation time (before flower vs. after initial fruit development) or differences in avian communities. Additionally, our inability to track the damage status of all apples on control trees (including those that feel from the tree) may have led to either an under- or overestimation of *C*. *pomonella* damage in the control trees. It is also conceivable that the netting protected arthropod predators such as spiders from birds, resulting in greater predation on *C*. *pomonella* and less fruit damage within exclosures [50]. Because it is unclear whether our findings are due to exclosure design or biological relevance, we are cautious in interpreting these results without further experimentation.

Several additional limitations of this study warrant further discussion. First, these data were collected over a single season and as such, we were not able to assess temporal variability in ecosystem services and disservices or bird habitat use. Furthermore, presence is not necessarily an indication of habitat quality [51], therefore, measuring bird survival and fecundity in apple orchards and adjacent habitat types should be a priority. Second, there may be variables that we did not measure that could have better explained habitat use as well as bird and *C*. *pomonella* damage. Future ecological research should investigate variables such as the proportion of undisturbed and human-altered habitat at multiple scales, the distance to anthropogenic features or population parameters such as the local density of birds and *C*. *pomonella* [22, 52]. Finally, farm-scale activities such as the volume and frequency of insecticide application, crop yield and cultural practices (e.g., fruit thinning) may relate to bird and *C*. *pomonella* presence and damage [14]. Because these farm-scale practices were impractical to incorporate into this study, we used farm ownership as a proxy variable to account for management practices.

Building on the scope and outcomes of this study, we highlight several priorities for future research. First, landscape-scale comparisons of bird habitat use in apple blocks and similarly-sized patches of undisturbed natural areas beyond the boundaries of the farm could provide further insight into the relative conservation value of organic apple orchards. Moreover, while there is a large body of literature that compares biodiversity in organic and conventional agroecosystems [53], future research regarding birds in apple orchards could specifically assess fruit damage and pest control as it relates to management practices. We expect the climate and fauna in and around orchards in other regions of the world are likely to vary substantially and relatively diverse sites could illuminate important variables affecting the nature and magnitude of animal-mediated ecosystem services.

Second, effects of bird activity can be quantified in monetary terms but non-monetary values may be just as important in influencing farm management practices. As an anecdotal example, the Lewis’s woodpecker may be valued by some farmers because it is attractive and charismatic. Several producers expressed a willingness to tolerate damage by this species relative to other, less charismatic birds because they enjoyed observing them on the farm (AMM personal observation). Social science methods such as producer surveys may help illuminate these non-monetary values and their influence on decision-making. Finally, birds may consume *C*. *pomonella* during life stages when the insects are not on the fruit (i.e., adult moths and pupae). Although beyond the scope of this study, it is also conceivable that birds damaging fruit could have been targeting *C*. *pomonella* larvae inside the apples. Investigating whether birds consume *C*. *pomonella* at all life stages, if birds preferentially attack or avoid insect-infested apples, and if there are long term benefits in yields would provide a more comprehensive assessment of their potential role in pest control and associated ecosystem services [54].

## Conclusions

We found that organic apple orchards in western Colorado provide habitat for a diversity of bird species, yet these birds did not appear to play a significant role in either fruit damage or pest control. Because rates of bird-induced fruit damage were low and attributed to a small subset of the avian community, organic orchards may provide bird habitat, even for species typically sensitive to human activities, without compromising production. Quantifying such synergies and tradeoffs between enhancing the habitat-value of agroecosystems and producing food is an emerging research priority [51].

## Supporting information

S1 Table Bird species detected in organic apple study sites. Common and scientific names of 59 species listed in American Ornithologists’ Union (AOU) order. Species were assigned as human-adapted (n = 40) or human-sensitive (n = 19) based upon the corresponding reference. When previous studies regarding species’ response to urbanization or housing density were not available, species were categorized based upon information in the Birds of North America species accounts. If multiple studies reported conflicting findings regarding species’ response to urbanization, the reference that was geographically closer to our study sites was used [27]. Detections were recorded through the following methods: TS (AB, AH) = transect survey (apple block or adjacent habitat including other crops, grass and shrub/tree habitat) and FO = foraging observations.
(DOCX)

S2 Table Occupancy model variables. Predictor variables included as covariates in single-season occupancy models for human-adapted and human-sensitive species for Ψ_1_ (psi; probability that a unit is occupied/used) and site and survey covariates for p_j_ (probability that species is detected in a unit in survey j, given presence). ArcGIS calculations made using ESRI [38].
(DOCX)

S3 Table Generalized linear mixed model predictor variables. Predictor variables included as covariates for comparison of *C*. *pomonella* damage related to bird access, apple block and apple characteristics.
(DOCX)

S4 Table Transect survey locations by species. Frequency counts for the locations where each human-adapted or human-sensitive species was detected. The adjacent shrub/tree habitat showed the greatest number of species (n = 43) while the adjacent grass habitat had the fewest (n = 21) and apple blocks were intermediate (n = 37). Encounter histories were used to estimate species richness using the second order jackknife estimator and to calculate Jaccard similarity indices.
(DOCX)

S5 Table Generalized linear mixed model selection results. AICc model selection results, investigating orchard and landscape characteristics affecting the proportion of *C*. *pomonella* damage as assessed in the exclosure experiment. Number of parameters (k), model weight, and log likelihood values are also reported. Apple block was included as a random effect and an observation-level random effect was included to account for overdispersion in all models. Models with substantial empirical support (i.e. ΔAICc value < 2) are shown in bold. However model weights indicated model uncertainty, therefore the strength and directionality of each predictor variable was model averaged from the entire model set.
(DOCX)
