# Supplementary material for: Bird use of organic apple orchards: Frugivory, pest control and implications for production
Source: PLoS One. 2017 Sep 14;12(9):e0183405. doi: 10.1371/journal.pone.0183405 (PMC5598930; doi:10.1371/journal.pone.0183405)
Supplement: S1 Table — Common and scientific names of 59 species listed in American Ornithologists’ Union (AOU) order. Species were assigned as human-adapted (n = 40) or human-sensitive (n = 19) based upon the corresponding reference. When previous studies regarding species’ response to urbanization or housing density were not available, species were categorized based upon information in the Birds of North America species accounts. If multiple studies reported conflicting findings regarding species’ response to urbanization, the reference that was geographically closer to our study sites was used [27]. Detections were recorded through the following methods: TS (AB, AH) = transect survey (apple block or adjacent habitat including other crops, grass and shrub/tree habitat) and FO = foraging observations. (DOCX) [file pone.0183405.s001.docx]

**S1 Table. Bird Species Detected in Organic Apple Study Sites.**

| **Species Name** | **Scientific Name** | **Human- adapted/ sensitive** | **Conservation Importance** | **Detection** |
| --- | --- | --- | --- | --- |
| mallard | *Anas platyrhynchos* | Adapted [[1](#_ENREF_1)] | Least Concern | TS (AH) |
| ring-necked pheasant | *Phasianus colchicus* | Sensitive [[2](#_ENREF_2)] | Least Concern | TS (AH) |
| turkey vulture | *Cathartes aura* | Sensitive [[3](#_ENREF_3)] | Least Concern | TS (AH) |
| Cooper's hawk | *Accipiter cooperii* | Adapted [[4](#_ENREF_4)] | Least Concern | TS (AH) |
| red-tailed hawk | *Buteo jamaicensis* | Adapted [[5](#_ENREF_5)] | Least Concern | TS (AH) |
| American kestrel | *Falco sparverius* | Adapted [[5](#_ENREF_5)] | Least Concern | TS (AB, AH), FO |
| killdeer | *Charadrius vociferus* | Adapted [[6](#_ENREF_6)] | Least Concern | TS (AH) |
| rock dove | *Columba livia* | Adapted [[6](#_ENREF_6)] | Least Concern | TS (AH) |
| Eurasian collared dove | *Streptopelia decaocto* | Adapted [[7](#_ENREF_7)] | Least Concern | TS (AB, AH) |
| white-winged dove | *Zenaida asiatica* | Adapted [[8](#_ENREF_8)] | Least Concern | TS (AH) |
| mourning dove | *Zenaida macroura* | Adapted [[6](#_ENREF_6)] | Least Concern | TS (AB, AH) |
| common nighthawk | *Chordeiles minor* | Adapted [[9](#_ENREF_9)] | Least Concern | TS (AB) |
| black-chinned hummingbird | *Archilochus alexandri* | Adapted [[3](#_ENREF_3)] | Least Concern | TS (AB, AH), FO |
| broad-tailed hummingbird | *Selasphorus platycercus* | Adapted [[10](#_ENREF_10)] | Least Concern | TS (AB, AH) |
| belted kingfisher | *Megaceryle alcyon* | Adapted [[3](#_ENREF_3)] | Least Concern | TS (AH) |
| Lewis's woodpecker | *Melanerpes lewis* | Sensitive [[11](#_ENREF_11)] | Watch List | TS (AB, AH), FO |
| downy woodpecker | *Picoides pubescens* | Sensitive [[3](#_ENREF_3)] | Least Concern | TS (AB, AH) |
| hairy woodpecker | *Leuconotopicus villosus* | Adapted [[12](#_ENREF_12)] | Least Concern | TS (AH) |
| northern flicker | *Colaptes auratus* | Adapted [[13](#_ENREF_13)] | Least Concern | TS (AB, AH), FO |
| western wood-pewee | *Contopus sordidulus* | Sensitive [[1](#_ENREF_1)] | Least Concern | TS (AB, AH), FO |
| least flycatcher | *Empidonax minimus* | Sensitive [[8](#_ENREF_8)] | Least Concern | TS (AH) |
| Say's phoebe | *Sayornis saya* | Sensitive [[14](#_ENREF_14)] | Least Concern | TS (AH) |
| western kingbird | *Tyrannus verticalis* | Sensitive [[3](#_ENREF_3)] | Least Concern | TS (AH) |
| Woodhouse’s scrub-jay | *Aphelocoma woodhouseii* | Adapted [[3](#_ENREF_3)] | Least Concern | FO |
| pinyon jay | *Gymnorhinus cyanocephalus* | Adapted [[15](#_ENREF_15)] | Watch List | TS (AH) |
| black-billed magpie | *Pica hudsonia* | Adapted [[16](#_ENREF_16)] | Least Concern | TS (AB, AH), FO |
| common raven | *Corvus corax* | Adapted [[17](#_ENREF_17)] | Least Concern | TS (AB, AH), FO |
| violet-green swallow | *Tachycineta thalassina* | Adapted [[18](#_ENREF_18)] | Least Concern | TS (AB, AH) |
| cliff swallow | *Petrochelidon pyrrhonota* | Adapted [[19](#_ENREF_19)] | Least Concern | TS (AH) |
| barn swallow | *Hirundo rustica* | Adapted [[1](#_ENREF_1)] | Least Concern | TS (AB, AH) |
| black-capped chickadee | *Poecile atricapillus* | Sensitive [[16](#_ENREF_16)] | Least Concern | TS (AB, AH) |
| bushtit | *Psaltriparus minimus* | Adapted [[1](#_ENREF_1)] | Least Concern | TS (AB, AH), FO |
| red-breasted nuthatch | *Sitta canadensis* | Adapted [[20](#_ENREF_20)] | Least Concern | TS (AB, AH) |
| house wren | *Troglodytes aedon* | Adapted [[16](#_ENREF_16)] | Least Concern | TS (AH), FO |
| American robin | *Turdus migratorius* | Adapted [[16](#_ENREF_16)] | Least Concern | TS (AB, AH), FO |
| European starling | *Sturnus vulgaris* | Adapted [[16](#_ENREF_16)] | Least Concern | TS (AB, AH) |
| cedar waxwing | *Bombycilla cedrorum* | Adapted [[21](#_ENREF_21)] | Least Concern | TS (AB, AH), FO |
| yellow warbler | *Setophaga petechia* | Sensitive [[3](#_ENREF_3)] | Least Concern | TS (AB, AH) |
| yellow-rumped warbler | *Setophaga coronata* | Adapted [[22](#_ENREF_22)] | Least Concern | TS (AH) |
| Wilson's warbler | *Cardellina pusilla* | Sensitive [[3](#_ENREF_3)] | Least Concern | TS (AB, AH) |
| green-tailed towhee | *Pipilo chlorurus* | Sensitive [[16](#_ENREF_16)] | Least Concern | FO |
| chipping sparrow | *Spizella passerina* | Sensitive [[17](#_ENREF_17)] | Least Concern | TS (AB, AH), FO |
| lark sparrow | *Chondestes grammacus* | Sensitive [[6](#_ENREF_6)] | Least Concern | TS (AB, AH) |
| white-crowned sparrow | *Zonotrichia leucophrys* | Adapted [[23](#_ENREF_23)] | Least Concern | TS (AB, AH) |
| dark-eyed junco | *Junco hyemalis* | Adapted [[24](#_ENREF_24)] | Least Concern | TS (AB, AH) |
| blue grosbeak | *Passerina caerulea* | Sensitive [[25](#_ENREF_25)] | Least Concern | TS (AB, AH), FO |
| lazuli bunting | *Passerina amoena* | Sensitive [[26](#_ENREF_26)] | Least Concern | TS (AB, AH), FO |
| red-winged blackbird | *Agelaius phoeniceus* | Adapted [[6](#_ENREF_6)] | Least Concern | TS (AB, AH), FO |
| western meadowlark | *Sturnella neglecta* | Sensitive [[6](#_ENREF_6)] | Least Concern | TS (AB, AH), FO |
| Brewer's blackbird | *Euphagus cyanocephalus* | Adapted [[26](#_ENREF_26)] | Least Concern | TS (AB, AH) |
| brown-headed cowbird | *Molothrus ater* | Adapted [[16](#_ENREF_16)] | Least Concern | TS (AB, AH) |
| Bullock's oriole | *Icterus bullockii* | Adapted [[26](#_ENREF_26)] | Least Concern | TS (AB, AH), FO |
| pine siskin | *Carduelis pinus* | Adapted [[27](#_ENREF_27)] | Least Concern | TS (AB, AH), FO |
| lesser goldfinch | *Spinus psaltria* | Sensitive [[17](#_ENREF_17)] | Least Concern | TS (AB, AH), FO |
| American goldfinch | *Spinus tristis* | Adapted [[26](#_ENREF_26)] | Least Concern | TS (AB, AH), FO |
| evening grosbeak | *Coccothraustes vespertinus* | Adapted [[28](#_ENREF_28)] | Watch List | TS (AH) |
| house sparrow | *Passer domesticus* | Adapted [[29](#_ENREF_29)] | Least Concern | TS (AH) |
| blue-gray gnatcatcher | *Polioptila caerulea* | Sensitive [[16](#_ENREF_16)] | Least Concern | FO |
| house finch | *Haemorhous mexicanus* | Adapted [[29](#_ENREF_29)] | Least Concern | TS (AB, AH), FO |

**S1 References**

1. Blair RB. Land use and avian species diversity along an urban gradient. Ecol Appl. 1996;6(2):506-19.

2. Giudice JH, Ratti JT. Ring-necked Pheasant: Phasianus Colchicus: American Ornithologists' Union; 2001.

3. Rottenborn SC. Predicting the impacts of urbanization on riparian bird communities. Biol Conserv. 1999;88(3):289-99.

4. Curtis OE, Rosenfield R, Bielefeldt J. Cooper's hawk (Accipiter cooperii) Ithaca: Cornell Laboratory of Ornithology; 2006. Available from: <http://birdsna.org>.

5. Berry ME, Bock CE, Haire SL. Abundance of diurnal raptors on open space grasslands in an urbanized landscape. Condor. 1998:601-8.

6. Lenth BA, Knight RL, Gilgert WC. Conservation value of clustered housing developments. Conserv Biol. 2006;20(5):1445-56.

7. Hansen AJ, Urban DL. Avian response to landscape pattern: the role of species' life histories. Landscape Ecol. 1992;7(3):163-80.

8. Chace JF, Walsh JJ. Urban effects on native avifauna: a review. Landscape Urban Plann. 2006;74(1):46-69.

9. Brigham R, Ng J, Poulin R, Grindal S. Common Nighthawk (Chordeiles minor) Ithaca: Cornell Laboratory of Ornithology; 2011.

10. Odell EA, Theobald DM, Knight RL. Incorporating ecology into land use planning: the songbirds' case for clustered development. Journal of the American Planning Association. 2003;69(1):72-82.

11. Vierling KT, Saab V, A., Tobalske BW. Lewis' Woodpecker (*Melanerpes lewis*) Ithaca: Cornell Laboratory of Ornithology; 2013. Available from: <http://birdsna.org>.

12. Jackson JA, Ouellet HR, Jackson BJ. Hairy Woodpecker (*Picoides villosus*) Ithaca: Cornell Lab of Ornithology; 2002. Available from: <http://birdsna.org>.

13. Wiebe KL, Moore WS, Poole A. Northern flicker (Colaptes auratus) Ithaca: Cornell Laboratory of Ornithology; 2008. Available from: <http://birdsna.org>.

14. Schukman JM, Wolf BO. Say's Phoebe (*Sayornis saya*) Ithaca: Cornell Laboratory of Ornithology; 1998. Available from: <http://birdsna.org>.

15. Balda RP. Pinyon Jay (*Gymnorhinus cyanocephalus*) Ithaca: Cornell Laboratory of Ornithology; 2002. Available from: <http://bna.birds.cornell.edu/bna/species/605>.

16. Odell EA, Knight RL. Songbird and Medium‐Sized Mammal Communities Associated with Exurban Development in Pitkin County, Colorado. Conserv Biol. 2001;15(4):1143-50.

17. Crooks KR, Suarez AV, Bolger DT. Avian assemblages along a gradient of urbanization in a highly fragmented landscape. Biol Conserv. 2004;115(3):451-62.

18. Brown CR, Knott AM, Damrose EJ. Violet-green swallow (*Tachycineta thalassina*) Ithaca: Cornell Lab of Ornithology; 2011. Available from: <http://birdsna.org>.

19. Brown CR, Brown MB. Cliff swallow (*Petrochelidon pyrrhonota*) Ithaca: Cornell Laboratory of Ornithology; 1995. Available from: <http://bna.birds.cornell.edu/bna/species/149>.

20. Ghalambor CK, Martin TE. Red-breasted nuthatch (*Sitta canadensis*) Ithaca: Cornell Laboratory of Ornithology; 1999. Available from: <http://birdsna.org>.

21. Witmer MC, Mountjoy DJ, Elliot L. Cedar waxing (*Bombycilla cedrorum*) Ithaca: Cornell Laboratory of Ornithology; 1997. Available from: <http://birdsna.org>.

22. Hunt P, Flaspohler DJ. Yellow-rumped warbler (*Setophaga coronata*) Ithaca: Cornell Laboratory of Ornithology; 1998. Available from: <http://birdsna.org>.

23. Chilton G, Baker MC, Barrentine CD, Cunningham MA. White-crowned sparrow (*Zonotrichia leucophrys*) Ithaca: Cornell Laboratory of Ornithology; 1995. Available from: <http://birdsna.org>.

24. Nolan V, Ketterson ED, Cristol DA, Rogers CM, Clotfelter ED, Titus RC, et al. Dark-eyed junco: (*Junco hyemalis*): Birds of North America; 2002.

25. Lowther PE, Ingold JL. Blue Grosbeak (Passerina caerulea) Ithaca: Cornell Laboratory of Ornithology; 2011. Available from: <http://birdsna.org>.

26. Maestas JD, Knight RL, Gilgert WC. Biodiversity across a rural land‐use gradient. Conserv Biol. 2003;17(5):1425-34.

27. Dawson WR. Pine Siskin (*Spinus pinus*) Ithaca: Cornell Laboratory of Ornithology; 2014. Available from: <http://birdsna.org>.

28. Gillihan SW, Byers B. Evening grosbeak: American Ornithologists' Union; 2001.

29. Bock CE, Bock JH, Bennett BC. Songbird abundance in grasslands at a suburban interface on the Colorado High Plains. Studies in Avian Biology. 1999;19:131-6.
