# Supplementary material for: Bird use of organic apple orchards: Frugivory, pest control and implications for production
Source: PLoS One. 2017 Sep 14;12(9):e0183405. doi: 10.1371/journal.pone.0183405 (PMC5598930; doi:10.1371/journal.pone.0183405)
Supplement: S2 Table — Predictor variables included as covariates in single-season occupancy models for human-adapted and human-sensitive species for Ψ1 (psi; probability that a unit is occupied/used) and site and survey covariates for pj (probability that species is detected in a unit in survey j, given presence). ArcGIS calculations made using ESRI [38]. (DOCX) [file pone.0183405.s002.docx]

**S2 Table. Occupancy Model Predictor Variables.**

| **Covariates used to model Ψ_1_** | | | | |
| --- | --- | --- | --- | --- |
| **Predictor Variable** | **Description** | **Measurement Method** | **Variable Type/Value** | **General Occupancy Predictions** |
| Management | Ownership/management of each farm; two of the three participating farms had similar management and were combined for this analysis | Farmer contacts | Categorical (Farm A, Farm B) | Vary by management |
| Habitat Locations | Where on the farm a transect was located | GPS locations and visual inspection of adjacent habitat type | Categorical (edge, interior) | + along edges;  - in interior |
| Edge-to-Area Ratio | The length (m) of the apple block edge to the area (m^2^) of the block containing the transect | ArcGIS edge length:block size area | Continuous (ratio) | + with increasing edge ratio |
| Size | The area (m^2^) of the apple block containing the transect | ArcGIS field calculation of polygons | Continuous (square meters) | - with increasing block size |
| **Covariates used to model p_j_** | | | | |
| **Survey Specific** | | | | |
| **Predictor Variable** | **Description** | **Measurement Method** | | **Variable Type/Value** |
| Observer | Observer who conducted the survey | Recorded by observer | | Categorical (0,1) |
| Wind | Wind speed category | Beaufort scale of wind speed as measured at time of survey | | Categorical (0 – 4) |
| Sky | Sky cover category | Scale of cloud cover and precipitation measured at time of survey | | Categorical (0 – 7) |
| Time | Time of day survey was conducted | Time of day (24 hour clock format) at which the survey was started | | Continuous |
| Temperature | Degrees Fahrenheit | Estimated within 5 degrees by observer assisted by a centrally-based thermometer | | Continuous |
| Date | Date survey was conducted | Recorded as MM/DD/YY by observer and transformed to the Julian day associated with the date the survey was conducted | | Continuous |
| Activity | Noise or activity associated with farm work (e.g., harvesting, thinning, etc.) near the transect | Described by observer and categorized if it was deemed loud enough to interfere with detections | | Categorical (0,1) |
| **Site Specific** | | | | |
| **Predictor Variable** | **Description** | **Measurement Method** | | **Variable Type/Value** |
| Effort | Effort (transect length) - transects were 50 m, 100 m or 200 m (combined transects in analysis) | Determined when transects were established | | Categorical (50, 100, 200) |
