# Supplementary material for: Bird use of organic apple orchards: Frugivory, pest control and implications for production
Source: PLoS One. 2017 Sep 14;12(9):e0183405. doi: 10.1371/journal.pone.0183405 (PMC5598930; doi:10.1371/journal.pone.0183405)
Supplement: S3 Table — Predictor variables included as covariates for comparison of C. pomonella damage related to bird access, apple block and apple characteristics. (DOCX) [file pone.0183405.s003.docx]

**S3 Table. Generalized Linear Mixed Model Predictor Variables.**

| **Predictor Variable** | **Description** | **Measurement Method** | **Variable Type (Value)** | **General Damage Predictions** |
| --- | --- | --- | --- | --- |
| Treatment | Apples enclosed in netting treatment to restrict bird access or unnetted control apples | Installed netting treatments or not | Binary (Treatment, Control) | + inside exclosure treatments |
| Habitat Location | Where in the apple block a surveyed tree was located | GPS locations and visual inspection of adjacent habitat type | Categorical (interior, edge) | - along edges where bird use is predicted to be greater |
| Apple Variety | The type of apple tree surveyed | Gathered from farmers | Categorical (Gala, Honeycrisp, Other (Akane, Golden Delicious, Granny Smith, Pristine, Rome, Zestar)) | Vary by variety |
| Management | Ownership/management of each farm; two of the three participating farms had similar management and were combined for this analysis | Farmer contacts | Categorical (Farm A, Farm B) | Vary by owner |
| Harvest Date | Julian day when apples were picked | Gathered from farmers or noted in field observations | Continuous (ordinal day number) | + with later harvest date |
