# Supplementary material for: Bird use of organic apple orchards: Frugivory, pest control and implications for production
Source: PLoS One. 2017 Sep 14;12(9):e0183405. doi: 10.1371/journal.pone.0183405 (PMC5598930; doi:10.1371/journal.pone.0183405)
Supplement: S4 Table — Frequency counts for the locations where each human-adapted or human-sensitive species was detected. The adjacent shrub/tree habitat showed the greatest number of species (n = 43) while the adjacent grass habitat had the fewest (n = 21) and apple blocks were intermediate (n = 37). Encounter histories were used to estimate species richness using the second order jackknife estimator and to calculate Jaccard similarity indices. (DOCX) [file pone.0183405.s004.docx]

**S4 Table. Transect Survey Locations by Species.**

| **Species Name** | **Apple** | **Adjacent Grass** | **Adjacent Shrub/Tree** |
| --- | --- | --- | --- |
| **Human-Adapted** |  |  |  |
| American kestrel | 9 | 3 | 19 |
| American robin | 77 | 14 | 27 |
| black-billed magpie | 5 | 7 | 21 |
| black-chinned hummingbird | 171 | 3 | 16 |
| Brewer's blackbird | 33 | 20 | 7 |
| common raven | 11 | 1 | 27 |
| Eurasian collared dove | 4 | 3 | 18 |
| European starling | 21 | 21 | 180 |
| house finch | 248 | 5 | 75 |
| mourning dove | 42 | 3 | 16 |
| northern flicker | 9 | 1 | 11 |
| red-winged blackbird | 2 | 2 | 9 |
| American goldfinch | 29 |  | 1 |
| barn swallow | 1 |  | 2 |
| brown-headed cowbird | 25 |  | 3 |
| Bullock's oriole | 7 |  | 14 |
| bushtit | 2 |  | 2 |
| dark-eyed junco | 1 |  | 3 |
| pine siskin | 3 |  | 2 |
| red-breasted nuthatch | 1 |  | 1 |
| white-crowned sparrow | 1 |  | 19 |
| belted kingfisher |  |  | 2 |
| broad-tailed hummingbird | 4 |  |  |
| cedar waxwing | 18 |  |  |
| common nighthawk | 1 |  |  |
| Cooper's hawk |  |  | 1 |
| evening grosbeak |  |  | 2 |
| house sparrow |  | 2 |  |
| house wren |  |  | 1 |
| killdeer |  | 2 |  |
| mallard |  |  | 1 |
| pinyon jay |  |  | 12 |
| violet-green swallow | 14 |  |  |
| white-winged dove |  |  | 1 |
| yellow-rumped warbler |  |  | 1 |
| **Human-Sensitive** |  |  |  |
| chipping sparrow | 66 | 4 | 7 |
| western meadowlark | 8 | 27 | 15 |
| western wood-pewee | 1 | 1 | 11 |
| black-capped chickadee | 1 |  | 2 |
| blue grosbeak | 19 |  | 6 |
| downy woodpecker | 2 |  | 2 |
| lark sparrow | 2 | 1 |  |
| lazuli bunting | 27 |  | 3 |
| lesser goldfinch | 34 |  | 5 |
| Lewis's woodpecker | 30 |  | 41 |
| western kingbird |  | 1 | 4 |
| Wilson's warbler | 1 |  | 1 |
| yellow warbler | 7 |  | 9 |
| least flycatcher |  |  | 1 |
| ring-necked pheasant |  | 1 |  |
| Say's phoebe |  | 1 |  |
| turkey vulture |  |  | 2 |
