# Supplementary material for: Bird use of organic apple orchards: Frugivory, pest control and implications for production
Source: PLoS One. 2017 Sep 14;12(9):e0183405. doi: 10.1371/journal.pone.0183405 (PMC5598930; doi:10.1371/journal.pone.0183405)
Supplement: S5 Table — AICc model selection results, investigating orchard and landscape characteristics affecting the proportion of C. pomonella damage as assessed in the exclosure experiment. Number of parameters (k), model weight, and log likelihood values are also reported. Apple block was included as a random effect and an observation-level random effect was included to account for overdispersion in all models. Models with substantial empirical support (i.e. ΔAICc value < 2) are shown in bold. However model weights indicated model uncertainty, therefore the strength and directionality of each predictor variable was model averaged from the entire model set. (DOCX) [file pone.0183405.s005.docx]

**S5 Table. Generalized Linear Mixed Model Selection Results.**

| **Model Names** | **k** | **AICc** | **ΔAICc** | **Weight** | **Log Likelihood** |
| --- | --- | --- | --- | --- | --- |
| **Treatment + Apple Variety + Management + (1\|Apple Block) + (1\|obs)** | **7** | **233.526** | **0.000** | **0.740** | **-108.86** |
| Treatment + Management + (1\|Apple Block) + (1\|obs) | 5 | 236.543 | 3.018 | 0.164 | -112.803 |
| Apple Variety + Management + (1\|Apple Block) + (1\|obs) | 6 | 239.414 | 5.888 | 0.039 | -113.040 |
| Treatment + Apple Variety + (1\|Apple Block) + (1\|obs) | 6 | 239.805 | 6.279 | 0.032 | -113.236 |
| Management + (1\|Apple Block) + (1\|obs) | 4 | 241.636 | 8.110 | 0.013 | -116.510 |
| Treatment + (1\|Apple Block) + (1\|obs) | 4 | 241.926 | 8.400 | 0.011 | -116.655 |
| Apple Variety + (1\|Apple Block) + (1\|obs) | 5 | 246.564 | 13.038 | 0.001 | -117.813 |
| 1 + (1\|Apple Block) + (1\|obs) | 3 | 248.498 | 14.972 | 0.000 | -121.067 |
